# Quality of life of patients with ADPKD**—**Toranomon PKD QOL study: cross-sectional study

**DOI:** 10.1186/1471-2369-14-179

**Published:** 2013-08-27

**Authors:** Tatsuya Suwabe, Yoshifumi Ubara, Koki Mise, Masahiro Kawada, Satoshi Hamanoue, Keiichi Sumida, Noriko Hayami, Junichi Hoshino, Rikako Hiramatsu, Masayuki Yamanouchi, Eiko Hasegawa, Naoki Sawa, Kenmei Takaichi

**Affiliations:** 1 Department of Nephrology, Toranomon Hospital, Tokyo, Japan

**Keywords:** Quality of life, Polycystic kidney disease, Dialysis, Total liver and kidney volume

## Abstract

**Background:**

The quality of life (QOL) of patients with autosomal dominant polycystic kidney disease (ADPKD) has not been investigated well. This study was performed to clarify the QOL of patients with ADPKD and to identify factors that affected their QOL.

**Methods:**

The present cross-sectional study is part of a prospective observational study on the QOL of ADPKD patients. Patients with ADPKD who were referred to Toranomon Hospital between March 2010 and November 2012 were enrolled. The short form-36 (SF-36) questionnaire and our original 12-item questionnaire were used to evaluate QOL. We analyzed the results of the questionnaire survey and then investigated correlations between QOL and clinical features.

**Results:**

A total of 219 patients (93 men and 126 women) were enrolled and their mean age was 55.1±10.8 years. There were 108 patients on dialysis. The SF-36 scores (PCS, MCS, and RCS) of all patients were significantly lower than the mean scores for the Japanese population. Stepwise multiple regression analysis demonstrated that Hb, serum Alb, ascites, and cerebrovascular disease all had a significant influence on the PCS, while mental disease had a significant influence on the MCS and serum Alb significantly influenced the RCS. The total liver and kidney volume (TLKV) and the dialysis status were not significantly associated with any of the SF-36 scores by multiple regression analysis, but TLKV was closely correlated with abdominal distention and distention had an important influence on QOL. Pain, sleep disturbance, heartburn, fever, gross hematuria, and anorexia also affected QOL, but these variables were not correlated with TLKV.

**Conclusions:**

Several factors influence QOL, so improving symptoms unrelated to TLKV as well as reducing abdominal distention can improve the QOL of ADPKD patients.

## Background

**Figure 1:**
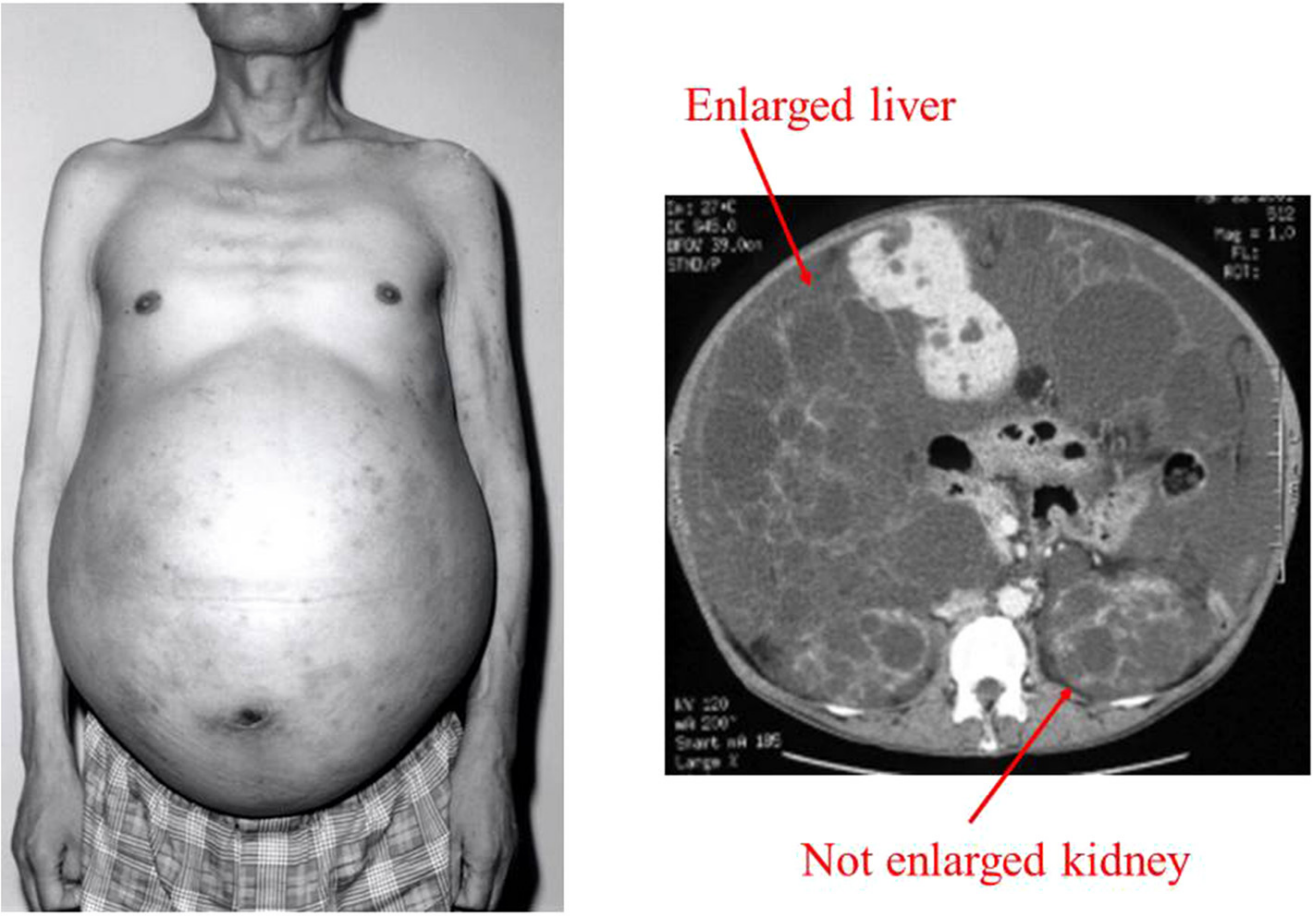
A non-dialysis ADPKD patient with massive hepatomegaly.

Autosomal dominant polycystic kidney disease (ADPKD) is a common disorder that occurs in approximately 1 in every 400 to 1000 live births [1,2]. It is estimated that less than one-half of ADPKD patients will be diagnosed during life because the disease is often clinically silent [1]. However, some patients with ADPKD develop massive enlargement of the kidneys and/or liver. We have performed renal transcatheter arterial embolization (TAE) in 797 symptomatic patients with renal enlargement and hepatic TAE in 325 symptomatic patients with hepatomegaly up to November 2012, and we have previously reported that TAE is an effective method of reducing the hepatic or renal volume [3-6]. Most of our patients who received renal or hepatic TAE had symptoms related to abdominal distension and their quality of life (QOL) was obviously impaired. It has been reported that ADPKD affects the physical condition of patients and also has an impact on mental health [7]. Pain is a common symptom of ADPKD and it has a detrimental effect on QOL [8,9], but the QOL of ADPKD patients and the factors influencing it have not been clarified. Recently, the TEMPO study showed that tolvaptan slows the increase of total kidney volume and the decline of renal function in patients with ADPKD [10], but it is still unknown what benefits are related to kidney volume and renal function. Rizk et al. evaluated the QOL of predialysis patients with ADPKD by the short form-36 (SF-36) questionnaire and they reported that such patients did not have worse QOL than the general population [11]. They also reported that the QOL of patients with a mean renal volume over 1000 cm^3^ was not significantly different from that of patients with a mean volume under 1000 cm^3^, but they did not assess the influence of hepatic volume. The average renal volume of the patients receiving renal TAE at our hospital was over 2000 cm^3^[3], and some of our predialysis patients also had severe hepatomegaly (Figure 1). We considered that the mean renal volume in Rizk’s study might have been too small to affect QOL and that hepatic volume should also be taken into account. Therefore, we started to investigate the QOL of ADPKD patients who were referred to our hospital for massive enlargement of the kidneys or liver, employing the SF-36 questionnaire and our original 12-item questionnaire to evaluate QOL. The SF-36 questionnaire is one of the most common methods of evaluating QOL worldwide, as well as in Japan [12-14]. Because mean SF-36 scores for the general Japanese population are available, it is easy to evaluate the QOL of patients by comparison with the general population. We also devised twelve questions of our own to evaluate the specific symptoms of patients with ADPKD.

In the present study, we employed these tools to clarify the QOL of ADPKD patients with massive enlargement of the kidneys and/or liver and to identify factors affecting their QOL.

## Methods

This was a cross-sectional study that was part of a prospective observational study on the QOL of patients with ADPKD. This study was reviewed and approved by the ethics committee of Toranomon Hospital in March 2010. Written informed consent was obtained from each participant before entry into the study, and also for publication of any accompanying images. A copy of the written consent for publication statement is available for review by the Editor of this journal. This study was registered with the University Hospital Medical Information Network (UMIN) as “Quality of life (QOL) of patients with autosomal dominant polycystic kidney disease (ADPKD): Influence of renal TAE or hepatic TAE on QOL”. All clinical information was obtained from the medial database of Toranomon Hospital.

### Patient population

All patients with ADPKD who were referred to Toranomon Hospital between March 2010 and November 2012 were enrolled in this study. Patients who were less than 20 years old, patients who received renal or hepatic TAE before March 2010, and patients who did not agree to participate in this research were excluded. Patients on CAPD were also excluded because abdominal distention might be influenced by their dialysis method. All enrolled patients met the criteria for diagnosis of ADPKD as defined by Ravine et al. [15]. Some patients underwent renal or hepatic TAE after being enrolled, but others did not undergo TAE up to November 2012.

### Assessment of QOL

Patients completed the self-administered questionnaires during their free time. They were requested to fill in the questionnaires while they were in a normal state. All patients who underwent renal or hepatic TAE completed the questionnaires before having the procedure. The questionnaires included the Short Form-36 questionnaire (SF-36) and our 12-item original questionnaire. The SF-36 contains 36 questions that evaluate eight dimensions: physical functioning, physical role function, body pain, general health perception, vitality, social function, emotional role function and mental health. The scores for these dimensions are then totalled to obtain a physical component summary score (PCS), a mental component summary score (MCS), and a role/social component summary score (RCS). These scores have been validated in various studies of multiple chronic illnesses and have been normalized for the general Japanese population (a mean value of 50 and an SD of 10). The mean age of the general Japanese population is 50.5 ± 15.9 years. We also devised 12 questions about specific symptoms of patients with ADPKD, such as abdominal distension, poor appetite, and sleep disturbance (Table 1). Each symptom was assigned a score of 1–4 or 1–5, with 1 being the most severe.

**Table 1:** Our original 12-item questionnaire

(Q1)	Can you cut your toenails by yourself?
(Q2)	Can you pick up something from the floor?
(Q3)	Do you feel abdominal distention?
(Q4)	Dou you suffer from heartburn?
(Q5)	How is your appetite?
(Q6)	How often do you take sleeping pills?
(Q7)	Dou you sleep well?
(Q8)	Do you snore?
(Q9)	How often do you take a purgative?
(Q10)	How often do you have constipation?
(Q11)	How often do you have fever of more than 38°C?
(Q12)	How often do you have gross hematuria?

### Clinical and laboratory assessments

Height and body weight were recorded when the subjects filled in the questionnaires. Dry weight was used as the body weight of dialysis patients. Body mass index (BMI) was calculated as the weight in kilograms divided by the square of the height in meters. All patients underwent laboratory tests while they were in their usual state and within one week before or after filling in the questionnaires. The past history was investigated from the medical records of Toranomon Hospital and all associated diseases with a medical diagnosis were identified from the records. With regard to the past history, we analyzed the influence on QOL of cardiovascular disease, cerebrovascular disease, cancer, mental disease, and diabetes mellitus.

### Imaging studies

Abdominal CT or MRI was routinely performed in all patients and images obtained within 3 months before or after entry into this study were used for evaluation of organ volumes. For all patients who received renal or hepatic TAE after being enrolled in this study, the abdominal CT or MRI studies obtained before TAE were used for evaluation of organ volume because it would have changed after TAE.

T1-weighted images (T1WI) and T2-weighted images (T2WI) obtained in the transverse and sagittal planes were employed for evaluation of organ size by MRI, which was performed with a 1.5-T apparatus (MagnetomAvanto, Siemens, Erlangen, Germany) and a phased-array body coil. CT scanning was performed with a 16-MDCT scanner (Aquilion 16, Toshiba).

Renal volume was calculated on CT scans using the formula for an ellipsoid: a*b* c*π/6, where a is the maximum length of the kidney and b and c are the maximum widths in the two transverse dimensions. Each CT scan obtained at a slice interval of 1 cm was analyzed using Synapse software (Fujifilm Company) and the hepatic area (cysts plus parenchyma) was measured. Then the total liver volume was calculated as the sum of all the hepatic areas, while the total liver and kidney volume (TLKV) was obtained by adding total liver volume and the kidney volumes calculated as mentioned above. The existence of ascites was assessed by two different readers who were specialists in nephrology and radiology.

### Statistical analysis

The mean PCS, MCS, RCS, and specific component scores were calculated for the overall study population. For quantitative discrete variables, differences between groups were assessed by using Student’s *t*-test or the Mann-Whitney U test.

The values of PCS, MCS, and RCS were compared between the patients and the general Japanese population by the one sample *t*-test. Correlations between PCS, MCS, or RCS and TLVK were assessed by univariate analysis.

Stepwise multiple regression analysis with forward elimination was used to analyze the factors related to PCS, MCS, and RCS. These three indicators of QOL were defined as the dependent variable, while age, sex, BMI, dialysis, duration of dialysis, ascites, TLKV, serum Alb, CRP, Cr, alkaline phosphatase (ALP), cholinesterase (ChE), Hb, cardiovascular disease, cerebrovascular disease, cancer, mental disease, and diabetes mellitus were defined as the explanatory variables. The TLKV of each patient was adjusted by the body surface areafor use in all analyses.

The answers to our 12-item questionnaire were expressed on an ordinal scale and correlations with the three SF-36 scores and TLKV were assessed by Spearman’s rank correlation coefficient analysis. Factor analysis was employed for multivariate analysis of the relations among the three SF-36 scores and each of our 12 questions. Promax rotation was used for factor analysis because the underlying factors were all considered to be correlated with each other to some extent. Because pain is a common symptom of ADPKD and has a detrimental influence on QOL [8,9], a question about pain was included in the SF-36 and the score was used to assess the relation between pain and other SF-36 scales or TLKV by Spearman’s rank correlation coefficient analysis. Factor analysis was also employed for multivariate analysis of the relations among pain and the three SF-36 scores. In all analyses, P<0.05 was considered to indicate significance, and all analyses were performed with the SPSS Statistics 18.0 statistical software package (SPSS Inc., Chicago, IL, USA).

## Results

A total of 219 patients were enrolled in this study, including 93 men and 126 women with a mean age of 55.1 ± 10.8 years. Among them, 108 patients were on hemodialysis (Table 2). Up to November 2012, 85 patients received renal TAE and 28 patients received hepatic TAE. Abdominal CT or MRI data for evaluation of TLKV were available in 173 patients.

**Table 2:** Profile of the enrolled patients

	**M/F**	**Age (years old)**	**BMI**	**Duration of HD (months)**	**Serum Alb (g/dL)**	**Serum ChE (IU/L)**	**Serum Cr (mg/dL)**	**Serum CRP (mg/dL)**	**Serum ALP (IU/L)**	**Hb (g/dL)**
All	93/126	55.1±10.8	22.7±4.1	74.5±61.8	3.48±0.41	230.8±68.6	2.08±1.89	0.33±0.69	247.2±126.3	11.48±1.71
Non-dialysis	44/67	52.3±11.9*	23.4±5.0†		3.71±0.32*	265.8±72.5*	2.08±1.89	0.21±0.39‡	240.6±116.5	12.22±1.72*
Dialysis	49/59	57.9±8.7*	21.9±2.4†	74.5±61.8	3.23±0.34*	201.2±48.6*		0.45±0.88‡	253.9±135.9	10.72±1.34*
**No. of patients with ascites (/patients available abdominal image data)**	**Liver volume (cm**^**3**^**)**	**Kidney volume (cm**^**3**^**)**	**Total liver and kidney volume (cm**^**3**^**)**	**No. of patients with cardiovascular disease**	**No. of patients with cerebrovascular disease**	**No. of patients with cancer**	**No of patients with mental disease**	**No of patients with diabetes**		
31/176	3573.1±3088.7	3908.6±2578.3	7457.2±3801.6	21	21	11	5	2		
10/72	3841.2±3550.9	3308.1±2566.2*	7073.6±4175.2‡	8	13	7	4	2		
21/104	3378.2±2620.3	4538.2±2487.9*	7935.1±3393.5‡	13	8	4	1	0		

*P<0.0001, †P<0.005, ‡P<0.05.

**Figure 2:**
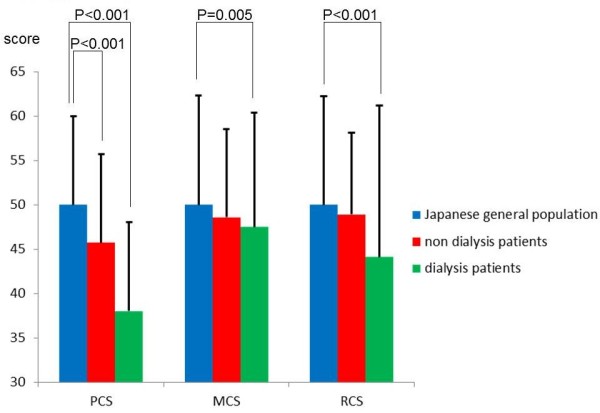
Comparison of the SF-36 scores (PCS, MCS, and RCS) for all enrolled patients, non-dialysis patients, dialysis patients, and the general Japanese population.

The three SF-36 scores (PCS, MCS, and RCS) of all patients were significantly lower than those of the general Japanese population (Figure 2). In addition, the PCS of patients aged from their 40s to 60s and the MCS and RCS of patients aged from their 50s to 60s were lower than those of the general Japanese population when analysis was performed by age group (Table 3). Among all non-dialysis patients, the PCS was significantly lower than the mean value for the general Japanese population (Figure 2). The PCS of non-dialysis patients from their 40s to 60s was also significantly lower than that of the general Japanese population (Table 3). Comparing non-dialysis patients with dialysis patients, the PCS was significantly lower for dialysis patients than non-dialysis patients, while the MCS and RCS did not differ between the two groups (Figure 2). Dialysis patients were older and had a lower BMI, lower Hb, lower serum ChE and Alb levels, and higher serum CRP level than non-dialysis patients (Table 2).

**Table 3:** Comparison of PCS, MCS, and RCS Comparison of PCS, MCS, and RCS between ADPKD patients and the general Japanese population

**Group**	**Item**	**Age group (decades)**	**No. of patients**	**Mean**	**SD**	**SEM**	P value*
All patients	PCS	30s	17	52.1	7.6	1.9	NS
		40s	52	44.2	13.4	1.9	<0.001
		50s	73	41.8	11.6	1.4	<0.001
		60s	58	36.2	14.9	2.0	<0.001
		70s	19	41.2	11.7	2.7	NS
	MCS	30s	17	52.7	10.5	2.5	NS
		40s	52	46.7	9.5	1.3	NS
		50s	73	47.6	7.7	0.9	<0.05
		60s	58	45.9	10.5	1.4	<0.001
		70s	19	55.7	8.2	1.9	NS
	RCS	30s	17	50.1	12.5	3.0	NS
		40s	52	47.6	15.5	2.2	NS
		50s	73	45.8	14.6	1.7	<0.001
		60s	58	46.0	17.3	2.3	<0.01
		70s	19	44.8	13.8	3.2	NS
Non-dialysis patients	PCS	30s	15	52.7	7.9	2.1	NS
		40s	34	47.6	13.0	2.2	<0.05
		50s	29	44.7	11.1	2.1	<0.05
		60s	23	37.9	17.3	3.6	0.001
		70s	10	43.4	11.8	3.7	NS
	MCS	30s	15	52.2	11.0	2.8	NS
		40s	34	47.0	10.2	1.7	NS
		50s	29	45.6	7.4	1.4	<0.01
		60s	23	48.4	11.0	2.3	0.05
		70s	10	57.4	4.5	1.4	NS
	RCS	30s	15	52.1	8.5	2.2	NS
		40s	34	47.9	14.0	2.4	NS
		50s	29	47.5	14.0	2.6	0.01
		60s	23	49.5	13.4	2.8	NS
		70s	10	50.1	11.9	3.8	NS
Dialysis patients	PCS	30s	2	47.5	0.6	0.5	NS
		40s	18	37.7	11.9	2.8	<0.001
		50s	44	40.0	11.7	1.8	<0.001
		60s	35	35.1	13.2	2.2	<0.001
		70s	9	38.8	11.8	3.9	NS
	MCS	30s	2	56.3	6.1	4.3	NS
		40s	18	45.9	8.2	1.9	NS
		50s	44	49.0	7.6	1.2	NS
		60s	35	44.2	9.9	1.7	<0.001
		70s	9	53.8	10.9	3.6	NS
	RCS	30s	2	35.2	31.4	22.2	<0.05
		40s	18	47.1	18.5	4.4	NS
		50s	44	44.6	15.0	2.3	<0.001
		60s	35	43.7	19.3	3.3	0.001
		70s	9	38.8	13.9	4.6	NS

* Comparison with the data of general Japanese population was analyzed in each age group.

SD: Standard Deviation, SE: standard error of the mean, NS: not significant.

**Figure 3:**
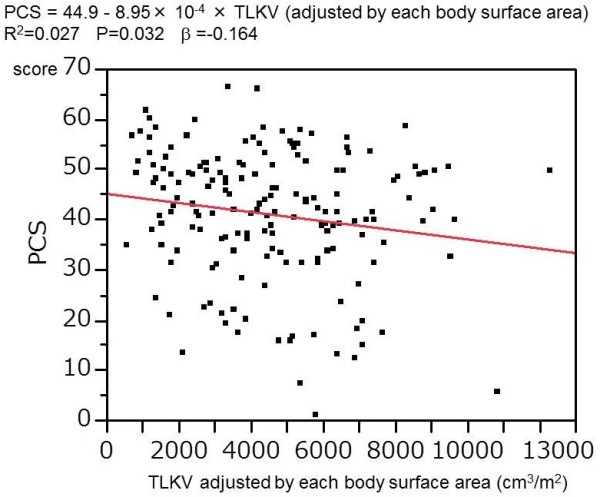
Univariate regression analysis of the relation between PCS and TLKV.

Univariate analysis revealed that PCS was correlated with TLKV, though MCS and PCS were not (Figure 3). Stepwise multiple regression analysis demonstrated that Hb serum Alb, ascites, and cerebrovascular disease were variables with a significant influence on PCS, while mental disease was the variable significantly influencing MCS and serum Alb was the only variable influencing RCS (Table 4). TLKV and dialysis were not significantly related to any of the SF-36 scores.

**Table 4:** Stepwise multiple logistic regression analysis of each SF-36 score

**Dependent variable**	**Explanatory variable**	**β**	**P value**	**Adjusted R**^**2**^
PCS	Alb	0.223	<0.01	0.187
	Hb	0.161	<0.05	
	Ascites	-0.214	<0.005	
	Cerebrovascular disease	-0.193	<0.01	
MCS	mental disease	−0.171	<0.05	0.023
RCS	Alb	0.247	0.001	0.055

β: Standardized regression coefficient.

Variables included in the model were age, sex, BMI, dialysis, dialysis duration, ascites, TLKV, Alb, CRP, Cr, ALP, ChE, Hb, cardiovascular disease, cerebrovascular disease, cancer, mental disease, and diabetes.

Among our original questions, Q1-7, Q11, Q12, and pain were significantly correlated with PCS by Spearman’s rank correlation analysis (Table 5), while Q1 and Q3 were correlated with TLKV. Thus, only Q1 and Q3 were correlated with both PCS and TLKV. According to factor analysis, PCS, Q3-5, and Q12 had high positive factor loadings, while PCS and Q11 had high negative factor loadings for the first factor (Table 6). Then PCS, Q1, and Q2 had high positive factor loadings for the second factor. Because the second factor has higher positive factor loadings than the first factor, Q1 and Q2 (with high positive loadings for the second factor) were considered to be better correlated with PCS than Q3. Since Q3-5, Q7, Q12, and pain were significantly correlated with MCS, Q3 was the only question correlated with both MCS and TLKV (Table 5). Factor analysis also showed that MCS, Q3-5, and Q12 had high positive factor loadings, while MCS and pain had high negative factor loadings for the first factor (Table 7). Since Q1-4, Q6, Q11, and pain were significantly correlated with RCS, only Q1 and Q3 were correlated with both RCS and TLKV (Table 5). Furthermore, RCS, Q7, and Q11 had high positive factor loadings, while RCS, Q5, and pain had high negative factor loadings for the second factor (Table 8).

**Table 5:** Spearman’s rank correlation coefficient (original questions vs. three SF-36 scores, and original questions vs. TLKV)

	** PCS**		** MCS**		** RCS**		** TLKV**	
**Question**	**r**_**s**_	**p**	**r**_**s**_	**P**	**r**_**s**_	**P**	**r**_**s**_	**P**
Q1	0.313	<0.001	0.127	NS	0.198	<0.005	−0.189	<0.05
Q2	0.366	<0.001	0.090	NS	0.337	<0.001	−0.148	NS
Q3	0.450	<0.001	0.357	<0.001	0.306	<0.001	−0.328	<0.001
Q4	0.235	0.001	0.388	<0.001	0.210	<0.005	0.044	NS
Q5	0.274	<0.001	0.379	<0.001	0.133	NS	−0.092	NS
Q6	0.189	<0.05	0.135	NS	0.179	<0.05	0.066	NS
Q7	0.290	<0.001	0.267	<0.001	0.126	NS	−0.072	NS
Q8	−0.009	NS	0.084	NS	−0.009	NS	0.077	NS
Q9	0.116	NS	0.037	NS	0.003	NS	−0.148	NS
Q10	0.007	NS	0.010	NS	0.004	NS	0.117	NS
Q11	0.162	<0.05	0.095	NS	0.163	<0.05	−0.030	NS
Q12	0.266	<0.001	0.163	<0.05	0.017	NS	−0.077	NS
Pain	−0.582	<0.001	−0.437	<0.001	−0.193	<0.005	−0.083	NS

r_s_: Spearman’s rank correlation coefficient, NS: not significant.

**Table 6:** Results of factor analysis of the PCS: Promax (oblique) rotated factor patterns

	**I**	**II**	**III**	**IV**	**V**
PCS	0.360	0.633	0.052	0.046	−0.059
Q1	−0.065	0.825	−0.012	0.041	−0.082
Q2	−0.045	0.847	0.135	−0.054	−0.006
Q3	0.739	0.273	−0.086	0.064	0.113
Q4	0.757	−0.122	0.191	−0.226	−0.112
Q5	0.843	−0.082	−0.309	0.015	−0.048
Q6	0.129	−0.109	−0.001	0.728	0.229
Q7	0.192	0.053	0.584	−0.124	0.024
Q8	0.007	−0.162	0.261	0.083	0.632
Q9	−0.214	0.145	−0.076	0.797	−0.176
Q10	0.051	−0.006	0.132	0.033	−0.805
Q11	−0.328	0.149	0.915	0.001	0.057
Q12	0.327	−0.269	0.380	0.290	−0.198
pain	−0.495	−0.287	−0.127	−0.076	−0.127
**Inter-factor correlations**					
I	-	0.225	0.424	0.206	0.037
II	0.225	-	0.159	0.095	−0.024
III	0.424	0.159	-	0.173	0.025
IV	0.206	0.095	0.173	-	−0.074
V	0.037	−0.024	0.025	−0.074	-

**Table 7:** Results of factor analysis of the MCS: Promax (oblique) rotated factor patterns

	**I**	**II**	**III**	**IV**	**V**
MCS	0.776	−0.194	−0.070	0.017	0.130
Q1	0.059	0.829	−0.026	0.077	−0.066
Q2	0.020	0.858	0.200	−0.037	−0.001
Q3	0.729	0.312	−0.056	0.089	0.066
Q4	0.721	−0.052	0.178	−0.222	−0.162
Q5	0.762	0.021	−0.243	0.014	−0.109
Q6	0.161	−0.084	−0.004	0.733	0.214
Q7	0.253	0.058	0.503	−0.107	0.015
Q8	−0.070	−0.139	0.398	0.053	0.563
Q9	−0.195	0.144	−0.056	0.792	−0.175
Q10	−0.023	−0.007	0.140	0.011	−0.825
Q11	−0.246	0.165	0.891	−0.027	0.045
Q12	0.248	−0.262	0.429	0.259	−0.263
pain	−0.531	−0.214	−0.200	−0.062	−0.121
**Inter-factor correlations**					
I	-	0.063	0.379	0.168	0.015
II	0.063	-	0.082	0.027	−0.060
III	0.379	0.082	-	0.143	−0.005
IV	0.168	0.027	0.143	-	−0.081
V	0.015	−0.060	−0.005	−0.081	-

**Table 8:** Results of factor analysis of the RCS: Promax (oblique) rotated factor patterns

	**I**	**II**	**III**	**IV**	**V**	**VI**
RCS	0.097	0.644	0.077	0.093	0.254	−0.223
Q1	0.103	−0.073	0.864	0.033	−0.054	−0.017
Q2	0.085	0.103	0.858	−0.083	−0.034	0.082
Q3	0.736	0.087	0.204	0.101	0.118	−0.023
Q4	0.678	0.294	−0.146	−0.201	−0.153	−0.086
Q5	0.848	−0.305	0.082	−0.026	−0.088	0.109
Q6	0.119	0.069	−0.193	0.760	0.155	0.043
Q7	0.130	0.680	−0.142	−0.048	−0.031	−0.122
Q8	0.050	−0.084	0.052	−0.059	0.230	0.944
Q9	−0.183	−0.082	0.145	0.789	−0.169	−0.122
Q10	0.026	−0.049	0.106	−0.035	−0.860	−0.291
Q11	−0.356	0.832	0.133	−0.030	−0.125	0.227
Q12	0.270	0.120	−0.142	0.192	−0.494	0.301
pain	−0.403	−0.328	−0.131	−0.074	−0.043	−0.053
**Inter-factor correlations**						
I	-	0.381	0.073	0.173	−0.051	0.091
II	0.381	-	0.218	0.146	−0.069	0.186
III	0.073	0.218	-	0.101	0.020	−0.112
IV	0.173	0.146	0.101	-	−0.098	0.084
V	−0.051	−0.069	0.020	−0.098	-	−0.183
VI	0.091	0.186	−0.112	0.084	−0.183	-

## Discussion

Rizk et al. reported that the SF-36 score of non-dialysis ADPKD patients was not significantly different from that of the general population. In the present study, however, the PCS of non-dialysis ADPKD patients was significantly lower than that of the general Japanese population. Our analysis stratified for age groups also showed that the PCS of non-dialysis patients from their 40s to 60s was significantly lower than that of the general Japanese population. This difference from the findings of Rizk et al. may have occurred because the mean kidney volume of the non-dialysis patients in our study was much larger than that of the patients they investigated. PCS was significantly lower in dialysis patients than non-dialysis patients, but age, nutritional status, and kidney volume were significantly different between the two groups.

It was reasonable that ascites and cerebrovascular disease were variables with a significant influence on PCS and mental disease was significantly associated with MCS. Serum Alb might reflect the nutritional status which is influenced by multiple factors, including the appetite, liver function, renal function, TLKV, and lifestyle. Then, it might not doubtful that Alb was significant variable for PCS and RCS. It might be the same reason for association Hb and PCS.

TLKV and dialysis were not significantly associated with any of the SF-36 scores by stepwise multiple regression analysis. However, univariate analysis revealed that PCS was correlated with TLKV, so TLKV seems to have an important influence on PCS. It seems that PCS was not only affected by abdominal distention (strongly correlated with TLKV), but also by several symptoms unrelated to TLKV. Accordingly, there should be multiple factors that influence the QOL of ADPKD in addition to TLKV, which is one of the reasons that TLKV was not significantly associated with PCS in multiple regression analysis. In addition, factor analysis revealed that Q1 and Q2 were more closely associated with PCS than Q3. Thus, the ability to cut toenails or to pick up something from the floor affected PCS more than abdominal distention. These abilities might be associated with muscular strength and balance as well as abdominal distension.

TLKV was also not significantly associated with MCS on multiple regression analysis. One of the reasons was that mental disease was a significant variable, but our 12-item questionnaire did not have any questions associated directly with mental disease. Symptoms associated with mental disease might have had higher positive factor loadings than abdominal distention. There was a weaker correlation between RCS and abdominal distension according factor analysis and Spearman’s rank correlation analysis. RCS might be more strongly affected by the social and family environment of each patient, but questions about the family and social environment were not included in this study.

As reported before, pain was an important factor for QOL because it was correlated with all of the SF-36 scores according to Spearman’s rank correlation analysis and factor analysis [8,9]. Sleep disturbance, heartburn, fever, gross hematuria and anorexia were also important factors for QOL, while snoring and constipation were not important factors. Though it is doubtful that gross hematuria influenced QOL, it might be related to fever and pain. Although pain, sleep disturbance, heartburn, fever, gross hematuria, and anorexia were all important for QOL, these variables were not correlated with TLKV. Thus, improving these factors unrelated to TLKV is considered to be important for elevating the QOL of ADPKD patients as well as reduction of abdominal distention.

Against our expectations, TLKV did not have a very strong influence on the QOL of ADPKD patients, although it was considered to have some influence on QOL. In order to reveal how TLKV affect SF-36 more clearly, we would need to study the changes of TLKV in each patient over time. Such a study about the changes of QOL in ADPKD patients after renal TAE is now ongoing and it will be able to reveal how SF-36 scores change when kidney volume is dramatically reduced by renal TAE. SF-36 did not reflect sensitively TLKV or abdominal distention in this study, so evaluation using SF-36 alone might not be suitable for assessing the symptoms of ADPKD patients. We will continue to use our original questions as well.

Against our expectation, dialysis did not have a significant influence on any of the SF-36 scores. One reason might be that QOL measured by SF-36 includes both symptoms affected by dialysis and symptoms unrelated to dialysis. In addition, the environment for dialysis patients (including techniques and medical expenses) is well developed in Japan and there are almost 20,000 patients who have been on hemodialysis for longer than 20 years [16]. This might have affected our results as well.

The limitations of this study are that it was based on a questionnaire and each answer was subjective. Also, only Japanese patients were included in this study, and there might be difference among ethnic groups and dialysis conditions in Japan might be different from those elsewhere in the world. There might also be other confounding factors that affect the QOL of ADPKD patients, but familial and social factors were not included in this study. Finally, the QOL of patients with ADPKD was mainly assessed with the SF-36 in this study, but it is uncertain how accurately this questionnaire reveals the actual situation of ADPKD patients.

## Conclusion

This study demonstrated that the mean PCS, MCS, and RCS of all patients were significantly lower than those of the general Japanese population. There was a significant difference of PCS between dialysis and non-dialysis patients, despite no significant differences of MCS and RCS. Even the PCS of non-dialysis patients was significantly lower than that of the general Japanese population. Stepwise multiple regression analysis demonstrated that Hb, serum Alb, ascites, and cerebrovascular disease were variables with a significant influence on PCS, while mental disease was the only significant factor for MCS and serum Alb was the only significant factor for RCS. TLVK and dialysis did not have a significant influence on any of the SF-36 scores according to stepwise multiple regression analysis. However, TLKV was well correlated with abdominal distention and abdominal distention was an important factor for QOL. Pain, sleep disturbance, heartburn, fever, gross hematuria, and anorexia were also important factors for QOL, but were not correlated with TLKV. Thus, QOL was considered to be a multifactorial construct and addressing symptoms unrelated to TLKV as well as abdominal distention is important for improving the QOL of ADPKD patients.

## Abbreviations

QOL: Quality of life; ADPKD: Autosomal dominant polycystic kidney disease; SF-36: The short form-36; TAE: Transcatheter arterial embolization; TLKV: Total liver and kidney volume; PCS: Physical component summary score; MCS: Mental component summary score; RCS: Role/social component summary score (RCS).

## Competing interests

All the authors declared no competing interest.

## Authors’ contributions

All co-authors carried out questionnaire survey on their patients, and collected questionnaires. YU conceived of the study, and participated in its design and coordination and helped to draft the manuscript. KT responsible person and participated in the design of the study. All co-authors read and approved the final manuscript.

## Pre-publication history

The pre-publication history for this paper can be accessed here:

http://www.biomedcentral.com/1471-2369/14/179/prepub
